# Coexistence of superconductivity and charge-density wave in the quasi-one-dimensional material HfTe_3_

**DOI:** 10.1038/srep45217

**Published:** 2017-03-24

**Authors:** Saleem J. Denholme, Akinori Yukawa, Kohei Tsumura, Masanori Nagao, Ryuji Tamura, Satoshi Watauchi, Isao Tanaka, Hideaki Takayanagi, Nobuaki Miyakawa

**Affiliations:** 1 Tokyo University of Science, Department of Applied Physics, Tokyo, 125-8585, Japan; 2 University of Yamanashi, Centre for Crystal Science and Technology, Yamanashi, 400-8511, Japan; 3 Tokyo University of Science, Department of Materials Science and Technology, Tokyo, 125-8585, Japan

## Abstract

We present the first experimental evidence for metallicity, superconductivity (SC) and the co-existence of charge density waves (CDW) in the quasi-one-dimensional material HfTe_3_. The existence of such phenomena is a typical characteristic of the transition metal chalcogenides however, without the application of hydrostatic pressure/chemical doping, it is rare for a material to exhibit the co-existence of both states. Materials such as HfTe_3_ can therefore provide us with a unique insight into the relationship between these multiple ordered states. By improving on the original synthesis conditions, we have successfully synthesised single phase HfTe_3_ and confirmed the resultant structure by performing Rietveld refinement. Using low temperature resistivity measurements, we provide the first experimental evidence of SC at ~1.4 K as well as a resistive anomaly indicative of a CDW formation at ~82 K. By the application of hydrostatic-pressure, the resistivity anomaly shifts to higher temperature. The results show that HfTe_3_ is a promising new material to help study the relationship between SC and CDW.

The coexistence of superconductivity (SC) and charge- and/or spin-density waves (CDW and/or SDW) is fundamental to our understanding behind the mechanism of high-*T_c_* SC and is one of the most significant challenges in condensed matter physics^1^. CDWs favor low dimensionality^2^ and materials such as the layered/chain-like transition metal di/trichalcogenides (MQ_2_/Q_3_) (where M = groups IV–VI transition metals and Q = sulfur, selenium and tellurium) have been collectively studied for such phenomena^3^. Examples include TaS_3_^4^ and NbS_3_^5^ which exhibit CDWs and TaSe_3_ which becomes a SC below 2.1 K^6^. Studies of SC in the MQ_2_/Q_3_ family often support a competitive relationship between the SC and CDW states; SC can be induced/enhanced by the suppression of the CDW. This is typically achieved by the application of hydrostatic pressure such as in the case of NbSe_2_^7^ and NbSe_3_^8^ or by chemical doping for Na_x_TaS_2_^9^ and Cu_x_TiSe_2_^10^. However, it is rare that the materials without chemical/physical modification exhibit the co-existence of both states. ZrTe_3_ is a material which shows the coexistence of a CDW at ~63 K and filamentary SC at 2 K^11^ as does NbSe_2_^3^. In the case of ZrTe_3_, by the application of pressure, intercalation of Cu^12^ and Ni^13^ or the substitution of Se at the Te site^14^, the CDW can be suppressed and bulk SC induced at ~5 K^15^. The electronic structure of ZrTe_3_ is unique amongst the MQ_3_ family owing to the strong contribution of the Te-Te p_σ*_ band at the vicinity of the Fermi level^16^, therefore the inter-chain interactions affects the electronic structure as well as the physical properties. Similar cross-chain interactions are absent in other members of the MQ_3_ family (when M = group IV transition metal and Q = S/Se)^17^. Of the MTe_3_ materials, HfTe_3_ is the only other material expected theoretically^18^,^19^. There are no known reports for TiTe_3_ nor Nb/TaTe_3_. However, by using the reaction conditions outlined by Brattås *et al*. we found that the successful synthesis of HfTe_3_^18^,^19^ was irreproducible. Therefore, although theoretical band structure calculations have predicted HfTe_3_ to be metallic^16^,^20^,^21^ there is currently no experimental confirmation. As far as the authors are aware, the available experimental data for HfTe_3_ include the original structural characterization^18^, and the determination of its basic magnetic properties (temperature-independent diamagnetism)^19^. In addition, it has been recently reported by scanning tunneling spectroscopy that Hf/HfTe_5_/HfTe_3_ films exhibited a superconducting gap-like spectra^22^. HfTe_3_ and ZrTe_3_ are iso-structural materials whose features raise the possibility that HfTe_3_ may also exhibit the coexistence of SC and CDW state. Therefore, it would be an important task to synthesize the high quality bulk compound, and to explore the aforementioned electrical phenomena.

By modifying the original synthesis conditions^18^,^19^, polycrystalline HfTe_3_ samples have been successfully synthesized. The crystal structure has been analyzed using Rietveld refinement and the first experimental evidence of metallicity in this material is reported. The resistivity data exhibits an anomaly suggestive of a CDW formation at ~82 K and subsequently zero resistivity below 2 K. By the application of hydrostatic pressure, the resistivity anomaly shifts to higher temperature. In addition, we note that HfTe_3_ is highly air-sensitive, where the behaviour of ρ-*T* characteristics changes from metallic to insulating upon exposure in air (See Supplementary information).

## Results and Discussion

### Key requirements to synthesise single phase HfTe_3_

Suitable reaction conditions to produce single phase HfTe_3_ crucially depend on the maximum reaction temperature^19^. During this investigation it has been found that a slow cooling rate is also a key requirement. In brief, the favoured phase was HfTe_2_ at a higher temperature range (≥530 °C) and HfTe_5_ at lower temperature regions (≤470 °C). As reported by Brattås *et al*., we confirmed that the sintering condition of c.a. 500 °C indeed favours the growth of the HfTe_3_ phase^18^. However, when rapid cooling from 500 °C (*e.g.* quenching in water) was applied^19^ the majority phase became HfTe_2_ together with unreacted tellurium. On the other hand, when slow cooling was performed (approx. −0.25 °C/h) until 470 °C after which the ampoules were cooled to room temperature at a rate of approx. −5 °C/h, then single phase HfTe_3_ could reproducibly be synthesised. The results suggest that HfTe_3_ primarily forms by reaction with the tellurium vapour upon cooling. If the reaction vessel is quenched, the solidification of the tellurium prevents its uptake and HfTe_2_ becomes the preferred phase. Namely, it is found that HfTe_3_ is the least thermodynamically stable phase within the Te-rich Hf alloys and as a result in order to inhibit the formation of trace amounts of HfTe_2_/HfTe_5_, it is necessary to control precisely both the sintering temperature and the cooling rate.

### Crystallographic analysis

**Figure 1:**
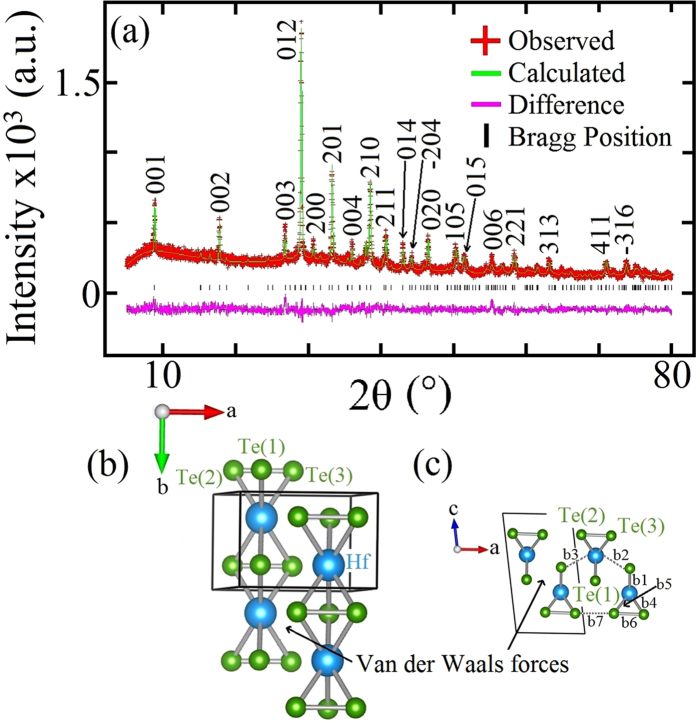
(**a**) Rietveld analysis of the PXRD results for HfTe_3_. (**b**) Crystal structure of HfTe_3_ revealing the anisotropic growth preferential along the *b*-axis. (**c**) Projection down the *b-*axis showing more clearly the separation of the chains, where the chains are weakly bonded by the Van der Waals forces. The positions of the three non-equivalent Te atoms are defined as Te(1), Te(2) and Te(3) and bond distances are indicated by b1-b7. The unit cell is indicated by the black lines.

Figure 1(a) shows the powder X-ray diffraction (PXRD) result for HfTe_3_ together with the result of the Rietveld refinement using ZrSe_3_ as a reference model^23^, where the result was consistent with the monoclinic crystal symmetry (space group P2_1_/m). Figure 1(b) represents the crystal structure of HfTe_3_ which is the pseudo-one-dimensional (1D) structure. As seen in Fig. 1(b), MQ_6_ trigonal prismatic units propagate along the *b*-axis resulting in chain-like anisotropic crystal growth. By projection down the *b*-axis it can be clearly seen how the chains are bonded together by Van der Waals forces (see Fig. 1(c)). Reasonable values of R_*wp*_ = 8.47%, R_*p*_ = 6.60% and χ^2^ = 1.544 were obtained. Refined lattice parameters of HfTe_3_, *a* = 5.8797(9) Å, *b* = 3.8999(9) Å, *c* = 10.0627(3) Å agreed with the previously reported values^19^. On the other hand, the angle *β* = 98.38(8)° showed a slight expansion from the originally reported angle of *β* = 97.98°^18^. The refinement results are summarized in Table 1. It was confirmed from X-ray fluorescence (XRF) results that the composition ratio of our HfTe_3_ was Hf:Te = 26:74 (at%).

### Coexistence of SC and CDW

**Figure 2:**
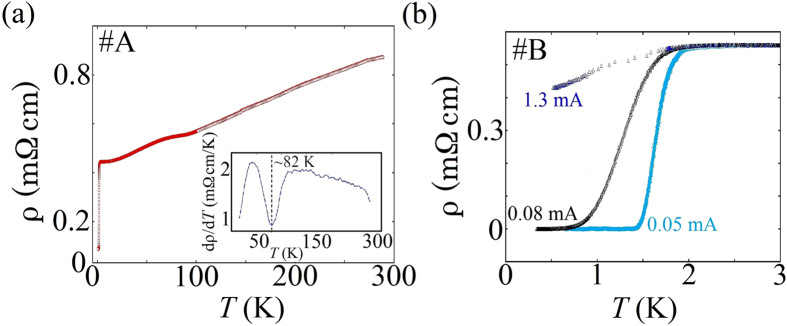
(**a**) Temperature dependence of the resistivity for HfTe_3_. Data show a hump-like feature at ~80 K together with and SC-like transition at 1.8 K. Inset shows that the resistivity anomaly occurs at approximately 82 K (sample #A). (**b**) Current dependency of the resistivity of HfTe_3_ below 3 K. *T*_c_^onset^ is approximately 1.8 K and *T*_c_^zero^ is reached c.a. 1.4 K for a current of 0.05 mA. By an increase in current, both the *T*_c_^onset^ and *T*_c_^zero^ show a shift to lower temperatures (sample #B).

Resistivity of non-air-exposed HfTe_3_ reproducibly exhibited metallic behaviour in the temperature range between 0.3 and 300 K as shown in Fig. 2(a). The residual resistivity ratio (RRR) defined as ρ(275 K)/ρ(4 K) is ~2.4, which is lower than that of single crystal ZrTe_3_^11^ but is larger than that of polycrystalline-ZrTe_3_^24^, in which the lower RRR value is thought to arise from strong grain boundary effects. Therefore the influence of grain boundaries is likely to play a role in the reduction of RRR. The inset of Fig. 2(a) shows the temperature derivative of the resistivity dρ/d*T* and reveals a resistivity anomaly at 82 K assumed to be indicative of a CDW formation, where the CDW formation temperature *T*_*CDW*_ is defined as the temperature at which dρ/d*T* exhibits a minimum. At *T*_*CDW*_ the CDW gap is developed and the resistance anomaly appears owing to a reduction in the density of states at *E*_*F*_ due to the CDW formation. Below 2 K, the resistivity showed a sharp drop exhibiting a SC transition at 1.8 K (*T*_c_^onset^) and reached zero (*T*_c_^zero^) at 1.45 K as can be clearly seen in Fig. 2(b). By increasing the applied current, a broadening of the SC transition was observed and it was accompanied by a downward shift in *T*_c_^onset^ and *T*_c_^zero^, whereas the normal state resistivity remains unchanged. The result suggests a weakening in the SC state as well as a decoupling of the Josephson junctions between individual SC grains of the polycrystalline material. *I*–*V* characteristics measured at *T* > *T*_c_ and *T* < *T*_c_ revealed ohmic and non-ohmic behaviour, respectively. N.B. In the present study, we observe that HfTe_3_ shows a rapid weakening of its metallic state within minutes of exposure in air (see Supplementary Fig. S1). This is likely the result of an insulating layer (such as tellurium oxides) forming around the individual grains of the polycrystalline material. The results emphasize that if one is to observe the intrinsic properties of HfTe_3_ any measurements must be conducted in the absence of air.

### Behaviour under high-pressure

**Figure 3:**
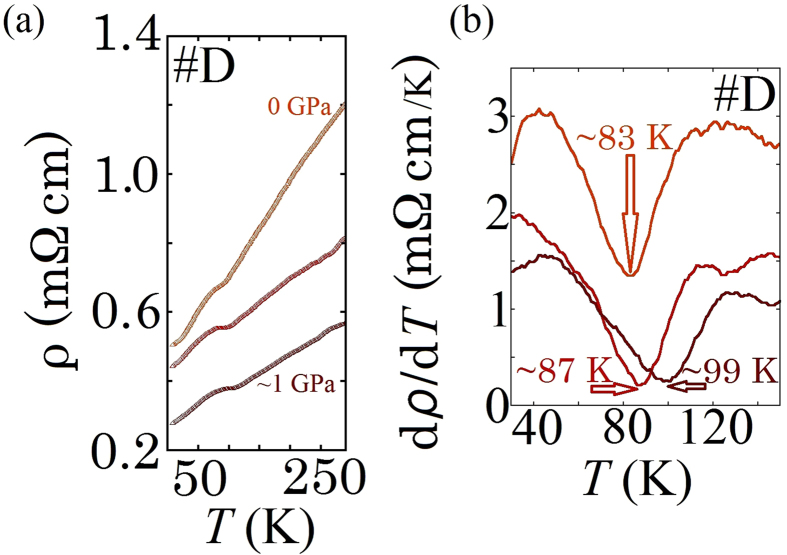
(**a**) Pressure dependence of resistivity at the range of *P* = 0–1 GPa. (**b**) dρ/d*T* in the range of 30–150 K as a function of pressure. The minimum of the dip structure shows a shift to higher *T* with increasing *P*. Color coding between (**a** and **b**) are matched to indicate the same pressure values (sample #D).

By the application of hydrostatic pressure (*P*), the resistivity anomaly gradually shifted to higher temperatures up to ~99 K for *P* approaching 1 GPa as shown in Fig. 3. Similar behaviour has been reported for ZrTe_3_ where in the case of an application of *P* ≤ 2 GPa the *T*_*CDW*_ was increased and the SC suppressed. At *P* ≥ 5 GPa the CDW was fully quenched and gave way to reemergent SC, where *T*_c_ increased to ~4.5 K when *P*~11 GPa^15^. In addition, in the case of HfTe_5_, SC appeared by applying *P*~5 GPa and a maximum *T*_c_ of 4.8 K was attained by applying at *P*~20 GPa^25^. This suggests the possibility that HfTe_3_ is likely to follow the same pattern as other members of the group IV-MTe_x_ alloys. Namely by further application in pressure, it is expected that the *T*_CDW_ will eventually be suppressed and *T*_c_ will be enhanced.

### Electronic structure

Studies regarding the electronic structure of HfTe_3_ are limited, but the issue is briefly reported by Felser *et al*. who determined an electronic structure similar to that of ZrTe_3_, *i.e.* a metallic state resulting from a large contribution of the Te *p*-bands at the Fermi level^16^. These characteristics are supported by later density of states (DOS) calculations^20^,^21^. ZrTe_3_ exhibits a multi-component Fermi surface with contributions from the Te forming quasi 1D electronic sheets at the boundary of the Brillion zone and from the Zr a 3D-hole character sheet around the Г point. The resultant nesting characteristics at the Fermi surface have been determined to be responsible for the CDW formation in ZrTe_3_^16^,^26^,^27^,^28^. If one considers the iso-structural/electronic relationship between HfTe_3_ and ZrTe_3_, it is likely that similar interchain interactions between neighbouring Te(2) and Te(3) atoms (see Fig. 1(c)) play a dominant role in the metallicity of HfTe_3_^16^ which in turn would give rise to the similar Fermi surface with nesting features reported for ZrTe_3_. However, it cannot be categorically asserted that the observed resistivity anomaly is due to a CDW formation from our results only. As in the case of ZrTe_3_, it would be necessary to confirm any coincidental low-temperature lattice distortions^29^ as well as to observe the features of the Fermi surface around the temperature of the anomaly^26^. However the similarities between HfTe_3_ and ZrTe_3_ in the electronic structure as well as the results of the temperature/pressure dependence of ρ are strong indication that the observed resistivity anomaly for HfTe_3_ is indeed the result of a CDW formation.

## Conclusion

In summary, we have established a reproducible synthesis method for high-quality polycrystalline HfTe_3_ and showed that it is an acutely air-sensitive material. By using high-quality HfTe_3_ we found that the quasi-1D HfTe_3_ is a novel SC with *T*_c_ ~ 1.4 K, and the SC state coexists with the CDW state which appears at *T*_CDW_~ 82 K. Furthermore, we provided the first accurate crystallographic data by Rietveld refinement of the PXRD of HfTe_3_.

## Methods

Single-phase polycrystalline HfTe_3_ samples have been prepared using standard chemical vapour transport techniques. Ground mixtures of a 1:3 molar ratio of powdered Hf and Te were sealed in silica ampoules under a vacuum of c.a. 3 mTorr using a rotary pump. The ampoules were heated in a box furnace using the reaction procedure described in the results and discussion. To prevent exposure to air, all sample preparation was conducted in an argon filled glovebox.

PXRD was carried out using a Rigaku Smartlab diffractometer in flat plate geometry with a Cu K_α_ radiation (λ = 1.54056 Å). Diffraction data were typically collected for 5° ≤ 2θ ≤ 80° with a 0.01° step size with scan times of 3 hours. Rietveld refinement was performed using the GSAS software package via the EXPGUI interface^30^,^31^. XRF analysis was performed using a JEOL JSX 1000 S ElementEye.

Resistivity measurements were performed on cold-pressed pellets using a standard four-terminal setup. Measurements for sample #A were performed between 0.3 and 300 K using an Oxford Instruments 3He cryostat, data were collected by an AC method using a low-noise amplifier and two lock-in amplifiers. Measurements for sample #B were performed by a DC method between 0.3 K and 15 K using a Quantum Design PPMS equipped with an adiabatic demagnetization refrigerator. The resistivity for Samples #C and #D were also measured by a DC method between 2 and 300 K using a closed cycle helium refrigerator. High-pressure resistivity measurements (up to 1 GPa) were performed using a BeCu/NiCrAl clamped piston-cylinder cell using Daphne 7373 as the fluid pressure transmitting medium with Pb employed as a manometer.

## Additional Information

**How to cite this article**: Denholme, S. J. *et al*. Coexistence of superconductivity and charge-density wave in the quasi-one-dimensional material HfTe_3_. *Sci. Rep.* **7**, 45217; doi: 10.1038/srep45217 (2017).

**Publisher's note:** Springer Nature remains neutral with regard to jurisdictional claims in published maps and institutional affiliations.

## Supplementary Material

Supplementary Information

## Figures and Tables

**Table 1:** Crystallographic data for HfTe_3_.

**Crystal system**	Monoclinic	**No. Observations**	7500,
**Space group**	P2_1_/m (No. 11)	**No. parameters**	31
***a* (Å)**	5.8797 (9)	**R_wp_**	0.0847
***b* (Å)**	3.8999 (9)	**R_p_**	0.066
***c* (Å)**	10.0627 (3)	**Goodness of fit, χ^2^**	1.544
***β* (°)**	98.38 (8)	**Temperature (K)**	295 K
***V* (Å^3^)**	228.28 (2)		
**Z**	2		
**Hf; 2*****e*** **(x, 1/4, z)**		**Te(1); 2*****e*** **(x, 1/4, z)**	
**x**	0.2590 (7)	**x**	0.7339 (7)
**z**	0.6881 (2)	**z**	0.5674 (3)
100 x U_iso_ (Å^2^)^*^	0.484	**100 x U_iso_ (Å^2^)**	0.484
**Occ**	1	**Occ**	1
**Te(2); 2*****e*** **(x, 1/4, z)**		**Te(3); 2*****e*** **(x, 1/4, z)**	
**x**	0.4173 (7)	**x**	0.9058 (6)
**z**	0.1625 (9)	**z**	0.1638 (3)
100 x U_iso_ (Å^2^)^*^	0.484	100 x U_iso_ (Å^2^)^*^	0.484
**Occ**	1	**Occ**	1
**Selected bond lengths (Hf-Te) (Å)**		**Selected bond lengths (Te-Te) (Å)**	
**Hf - Te(1) x2 (b1)^†^**	3.074 (6)	**Te(2) - Te(3) x1 (b6)^†^**	2.870 (13)
**Hf - Te(1) x1 (b2)^†^**	3.106 (8)	**Te(2) - Te(3) x1 (b7)^†^**	3.010 (13)
**Hf - Te(1) x1 (b3)^†^**	3.106 (6)		
**Hf - Te(2) x2 (b4)^†^**	3.062 (7)		
**Hf - Te(3) x2 (b5)^†^**	2.843 (6)		

^*^Isotropic displacement factors were constrained during refinement. ^†^Refer to Fig. 1(c).

## References

[b1] Gabovich A. M., Voitenko A. I. & Ausloos M. Charge- and spin-density waves in existing superconductors: competition between cooper pairing and peirels or excitonic instabilities. Phys. Rep. 367, 583–709 (2002).

[b2] Grüner G. The dynamics of charge-density waves. Rev. Mod. Phys. 60, 1129–1178 (1988).

[b3] Wilson J. A., Di Salvo F. J. & Mahajan S. Charge-density waves and superlattices in the metallic layered transition metal dichalcogenides. Adv. Phys. 24, 117–201 (1975).

[b4] Ido M., Tsutsumi K., Sambongi T. & Mori N. Pressure dependence of the metal-semiconductor transition in TaS_3_. Solid State Comm. 29, 399–402 (1979).

[b5] Wang Z. . Charge-density-wave transport above room temperature in a polytype of NbS_3_. Phys. Rev. B. 40, 11589 (1989). 10.1103/physrevb.40.11589 9991758

[b6] Yamamoto M. Superconducting properties of TaSe_3_. J. Phys. Soc. Jpn. 45, 431–438 (1978).

[b7] Suderow H., Tissen V. G., Brison J. P., Martínez J. C. & Viera S. Pressure induced effects on the fermi surface of superconducting 2H-NbSe_2_. Phys. Rev. Lett. 95, 117006 (2005). 16197038 10.1103/PhysRevLett.95.117006

[b8] Núñez Reguerio M., Mignot J. M. & Castello D. Superconductivity at high pressure in NbSe_3_. Europhys. Lett. 18, 53–57 (1992).

[b9] Fang L. . Fabrication and superconductivity of Na_x_TaS_2_ crystals. Phys. Rev. B. 72, 014534 (2005).

[b10] Morosan E. . Superconductivity in Cu_x_TiSe_2_. Nature Phys. 2, 544–550 (2006).

[b11] Takahashi S., Sambongi T., Brill J. W. & Roark W. Transport and elastic anomalies in ZrTe_3_. Solid State Comm. 49, 1031–1033 (1984).

[b12] Zhu X., Lei H. & Petrovic C. Coexistence of bulk superconductivity and charge density wave in Cu_x_ZrTe_3_. Phys. Rev. Lett. 106, 246404 (2011). 21770585 10.1103/PhysRevLett.106.246404

[b13] Lei H., Zhu X. & Petrovic C. Raising T_c_ in charge density wave superconductor ZrTe_3_ by Ni intercalation. EPL. 95, 17011 (2011).

[b14] Zhu X. . Superconductivity and charge density wave in ZrTe_3-x_Se_x_. Sci. Rep. 6, 26974 (2016). 27253150 10.1038/srep26974 PMC4890587

[b15] Yomo R., Yamaya K., Abliz N., Hedo M. & Uwatoko Y. Pressure effect on competition between charge density wave and superconductivity in ZrTe_3_: appearance of pressure-induced reentrant superconductivity. Phys. Rev. B. 71, 132508 (2005).

[b16] Felser C., Finckh E. W., Kleinke F., Rocker F. & Tremel W. Electronic properties of ZrTe_3_. J. Mater. Chem. 8, 1787–1798 (1998).

[b17] Srivastava S. K. & Avasthi B. N. Preparation, structure and properties of transition metal trichalcogenides. J. Mater. Sci. 27, 3693–3705 (1992).

[b18] Brattås L. & Kjekshus A. The non-metal rich region of the Hf-Te system. Acta Chem. Scand. 25, 2783–2784 (1971).

[b19] Brattås L. & Kjekshus A. On the properties of compounds with the ZrTe_3_ type structure. Acta Chem. Scand. 26, 3441–3449 (1972).

[b20] Abdulsalam M. & Joubert D. P. Structural and electronic properties of MX_3_ (M = Ti, Zr and Hf; =S, Se, Te) from first principles calculations. Eur. Phys. J. B. 88, 177 (2015).

[b21] Li M., Dai J. & Cheng-Zeng X. Tuning the electronic properties of transition-metal trichalcogenides *via* tensile strain. Nanoscale 7, 15385–15391 (2015). 26332584 10.1039/c5nr04505c

[b22] Wang Y. . Spontaneous formation of a superconductor-topological insulator-normal metal layered heterostructure. Adv. Mater. 28, 5013–5017 (2016). 27087261 10.1002/adma.201600575

[b23] Furuseth S., Brattås L. & Kjekshus A. On the crystal structures of TiS_3_, ZrS_3_, ZrSe_3_, ZrTe_3_, HfS_3_ and HfSe_3_. Acta Chem. Scand. 29, 623–631 (1975).

[b24] Yadav C. S. & Paulose P. L. Superconductivity at 5.2 K in ZrTe_3_ polycrystals and the effect of Cu and Ag intercalation. J. Phys.: Condens. Matter. 24, 235702 (2012). 22585457 10.1088/0953-8984/24/23/235702

[b25] Qi Y. . Pressure-driven superconductivity in the transition-metal pentatelluride HfTe_5_. Phys. Rev. B. 94, 054517 (2016).

[b26] Kubo Y. . Electron momentum density in the low dimensional layered system ZrTe_3_. J. Phys. Soc. Jpn. 76, 064711 (2007).

[b27] Stöwe K. & Wagner F. R. Crystal structure and calculated electronic band structure of ZrTe_3_. J. Solid State Chem. 138, 160–168 (1998).

[b28] Hoesch M. . Splitting in the fermi surface of ZrTe_3_: a surface charge density wave system. Phys. Rev. B. 80, 075423 (2009).

[b29] Eaglesham D. J., Steeds J. W. & Wilson J. A. Electron microscope study of superlattices in ZrTe_3_. J. Phys. C: Solid State Phys. 17, L697–L698 (1984).

[b30] Larson A. C. & von Dreele R. B. The General Structure Analysis system. Los Alamos National Laboratories, Los Alamos, NM (2000).

[b31] Toby B. H. EXPGUI, a graphical user interface for GSAS. J. Appl. Crystallogr. 34, 210–213 (2001).

