# Supplementary material for: Coexistence of superconductivity and charge-density wave in the quasi-one-dimensional material HfTe3
Source: Sci Rep. 2017 Mar 24;7:45217. doi: 10.1038/srep45217 (PMC5364548; doi:10.1038/srep45217)
Supplement: Supplementary Information [file srep45217-s1.pdf]

Coexistence of superconductivity and charge-density wave in the quasi-one-dimensional material $HfTe_3$

Saleem J. Denholme[1,*], Akinori Yukawa[1], Kohei Tsumura[1], Masanori Nagao[2], Ryuji Tamura[3], Satoshi Watauchi[2], Isao Tanaka[2], Hideaki Takayanagi[2] and Nobuaki Miyakawa[1,*]

[1]Tokyo University of Science, Department of Applied Physics, Tokyo, 125-8585, Japan

[2]Tokyo University of Science, Department of Materials Science and Technology, Tokyo, 125-8585, Japan

[3]University of Yamanashi, Interdisciplinary Graduate School of Medicine and Engineering, Tokyo, 400-8511, Japan

[*]sdenholme@rs.tus.ac.jp

[*]miyakawa@rs.tus.ac.jp

Supplementary information

Air sensitivity. We conducted a systematic investigation to analyse the effect air exposure has on the physical properties of $HfTe_3$. $HfTe_3$ shows a rapid weakening of its metallic state within minutes of exposure in air as shown in Fig. S1(a). No change in lattice constants was observed from the PXRD measurement of the 1h air exposed samples as shown in Fig. S1(b). However, PXRD patterns of samples exposed in air for more than one week showed the formation of partially amorphous state as well as degradation in peak intensity. From Fig. S1(c), it is found that the dip in $d\rho/dT$ relating to the resistivity anomaly deepened as a function of time exposed in air, but there was no shift in the dip position. On the other hand, $HfTe_3$ stored in an argon atmosphere/vacuum was stable. These results suggest that the insulting behaviour is a non-intrinsic property and a result of an insulating layer forming around the individual grains of the polycrystalline material. It is currently unclear why $HfTe_3$ degrades so easily although the Zr equivalent does not. However it is likely related to the polycrystallinity; almost all $ZrTe_3$ studies have been performed on single-crystalline materials. It had been previously reported that all members of the Zr/Hf-telluride family exhibit oxidative degradation.[1] Single crystal $HfTe_2$ [2] as well as powdered $ZrTe_2$ [3] are also evidently air-sensitive materials but similar observations have not been reported for $ZrTe_3$. The increased ionicity of Hf in comparison to Zr may also play a role. Further investigation into the thermodynamic stability of the Hf-Te system is required if one is to gain a better understanding of this system.

1. Fjellvåg, H., Furuseth, S., Kjekshus, A. & Rakke, T. Low-temperature oxidative degradation of low-dimensional zirconium and hafnium tellurides. *Solid State Comm*. **63,** 293-297 (1987).
2. Smeggil, J.G. & Bartram, S. The preparation and X ray characterization of $HfTe_{2-x}$, x = 0.061. *J. Solid State Chem.* **5,** 391-394 (1972).
3. Aoki, Y., Sambongi, T., Levy, F. & Berger, H. Thermopower of $HfTe_2$ and $ZrTe_2$. *J. Phys. Soc. Jpn*. **65,** 2590-2593 (1996).

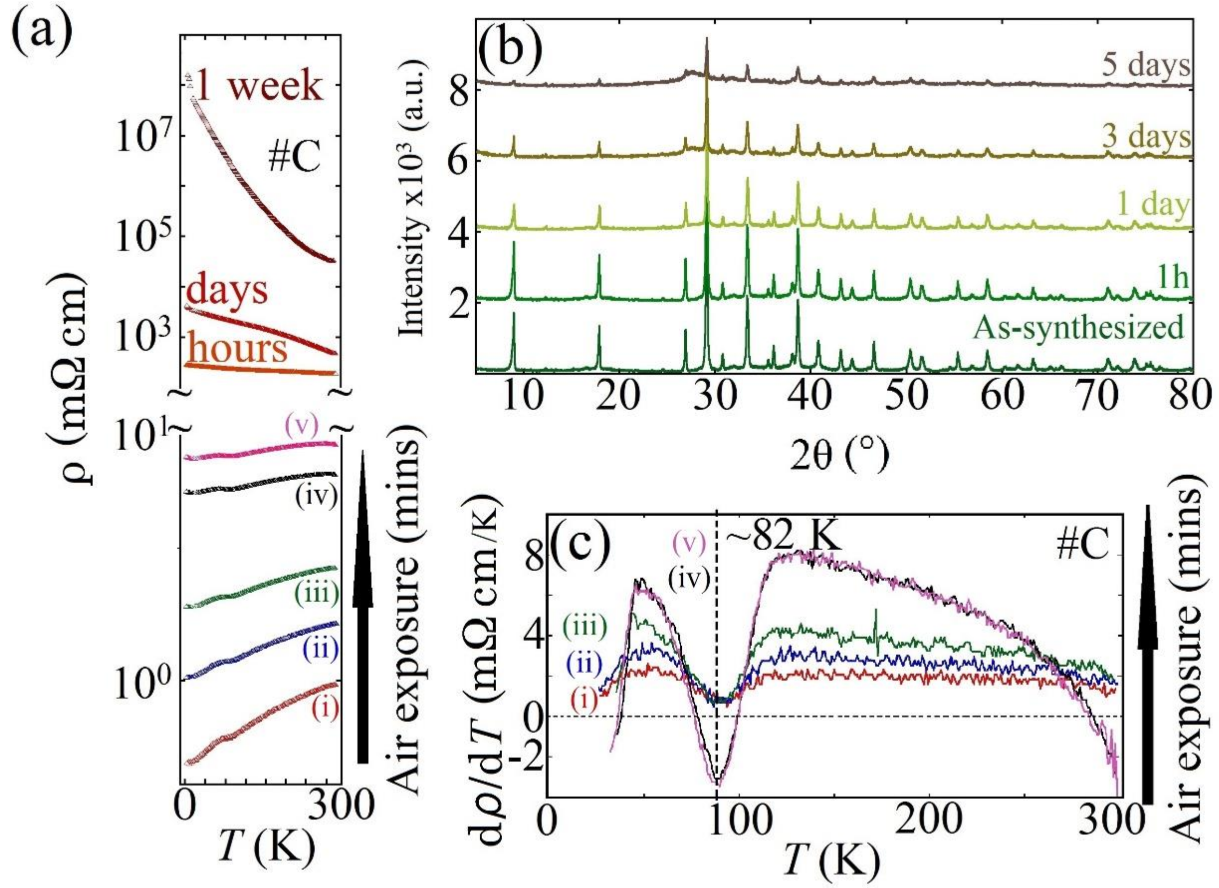

Figure S1. (a) Semi-log plot of resistivity of $HfTe_3$ as a function of exposure time in air. (i)~ 1min (ii)~ 5mins (iii)~ 10mins (iv)~ 20mins (v)~ 40mins (sample #C). (b) PXRD of an air-exposed sample up to five days. Intensity of peaks gradually decreases and an amorphous-like hump feature begins to appear at 20-30° range. (c) Characteristics of dρ/dT for the resistivity data (i) - (v) under air exposure in $HfTe_3$ (sample #C).
